# Plant health status effects on arbuscular mycorrhizal fungi associated with *Lavandula angustifolia* and *Lavandula intermedia* infected by Phytoplasma in France

**DOI:** 10.1038/s41598-020-77240-6

**Published:** 2020-11-20

**Authors:** Marie- Noëlle Binet, Camille Marchal, Justine Lipuma, Roberto A. Geremia, Olivier Bagarri, Bert Candaele, Delphine Fraty, Benjamin David, Sophie Perigon, Viviane Barbreau, Bello Mouhamadou

**Affiliations:** 1 Laboratoire d’Écologie Alpine, CNRS UMR 5553, Université Grenoble Alpes, CS 40700, 38058 Grenoble Cedex 09, France; 2 MYCOPHYTO, 50 Avenue de la Plaine, 06250 Mougins, France; 3 Université Européenne des Senteurs et des Saveurs, Couvent des Cordeliers, 04300 Forcalquier, France; 4 Centre Régionalisé Interprofessionnel d’Expérimentation en Plantes Aromatiques et Médicinales, Lieu-dit Les Quintrands, Route de Volx, 04100 Manosque, France; 5 Collège Henri Wallon, 17 rue Henri Wallon, 38400 Saint Martin d’Hères, France

**Keywords:** Ecology, Ecology, Environmental sciences

## Abstract

We investigated root communities of arbuscular mycorrhizal fungi (AMF) in relation to lavender (*Lavandula angustifolia*) and lavandin (*Lavandula intermedia*) health status from organic and conventional fields affected by Phytoplasma infection. The intensity of root mycorrhizal colonization was significantly different between diseased and healthy plants and was higher in the latter regardless of agricultural practice. This difference was more pronounced in lavender. The root AMF diversity was influenced by the plant health status solely in lavender and only under the conventional practice resulting in an increase in the AMF abundance and richness. The plant health status did not influence the distribution of root AMF communities in lavandin unlike its strong impact in lavender in both agricultural practices. Finally, among the most abundant molecular operational taxonomic units (MOTUs), four different MOTUs for each plant species were significantly abundant in the roots of healthy lavender and lavandin in either agricultural practice. Our study demonstrated that the plant health status influences root colonization and can influence the diversity and distribution of root AMF communities. Its effects vary according to plant species, can be modified by agricultural practices and allow plants to establish symbiosis with specific AMF species.

## Introduction

Arbuscular mycorrhizal fungi (AMF, Glomeromycota) are obligate symbiotic fungi which colonize roots of the majority of land plants, including the most important food crops^1^. They provide essential ecosystem services by improving plant nutrient availability, plant diversity and soil structure formation^2^. They also enhance plant resistance to abiotic stresses^3^ as well as to biotic stresses^4,5^. Many studies have shown the important role of AMF against a wide range of phytopathogens resulting in a reduction or suppression of plant disease symptoms^6^. These remarkable properties make the AMF key players in cropping systems to increase crop yields and plant defenses within the framework of sustainable agriculture. Studying the diversity and the drivers shaping AMF communities in roots of agricultural crops could help developing strategies involving these fungi in relation to plant health^7^.

Although scarcely studied, plant health status seems to play an important role on AMF community structure and diversity. For instance, clear relationship between root-associated fungal communities’ composition and plant health status in *Pisum sativum* has been reported^7^. They found that AMF, described as able to strengthen the defense of plants against phytopathogenic agents or improving their fitness, were more abundant and diverse in healthy pea plants. Likewise, an important shift in diversity and composition of root AMF communities when comparing healthy to diseased plants in Salix bioenergy plantations was shown^8^. In both studies, the effects of plant health status were investigated on sites characterized by the same mode of agricultural practices. To our knowledge there is no information on the importance of the plant health status on root AMF communities associated to plant crops under different agricultural systems such as conventional or organic practices. Both practices are often increasingly developed to improve crop yields and are also reported to affect AMF communities^9^. For example, organic farming systems via the crop rotation, the cover cropping or a lack of synthetic pesticides and inorganic fertilizers promote root colonization and abundance of spores in root systems maintaining alongside a greater diversity of AMF communities in the soil^10^. Conversely, in conventional farming the negative effects of high input cropping systems or conventional flooding on AMF communities have been reported due probably to the disturbance of the hyphal networks they provoke^11,12^. However, since conflicting results have also been described, showing very weak effects or even the lack of effects of agricultural practices on the community composition of AMF in roots^13,14^, we suggested that possible interactive effects with other biotic or abiotic factors such as the host plant health status could exist, thereby impacting AMF communities.

The purpose of this article was to study the responses of AMF to plant health status on two aromatic plants, *L. angustifolia* and *L. intermedia* cultivated under different agricultural practices in south-eastern France. In these lavender agricultural systems, the yellow decline of lavender caused by the stolbur phytoplasma ‘*Candidatus Phytoplasma solani*’ reduced seriously plant productivity^15^. Otherwise, different agricultural practices such as conventional or organic farming were used for both *L. angustifolia* and *L. intermedia* so as to find a strategy to reduce the impact of the disease. In both practices, diseased and healthy plants coexisted. The latter are likely the result of either the absence of contact with phytoplasma or biotic interactions such as the establishment of a mycorrhizal symbiosis with specific AMF communities able to suppress plant damage as reported elsewhere^16–19^. By studying the root AMF communities associated to the healthy and diseased lavender and lavandin, we tested the hypothesis that the AMF colonization, the diversity and the composition of root AMF communities could be influenced by the plant health status. We also sought to determine whether these plant health status effects varied according to the agricultural practice and to the plant species identity.

## Results

### AMF root colonization

**Figure 1:**
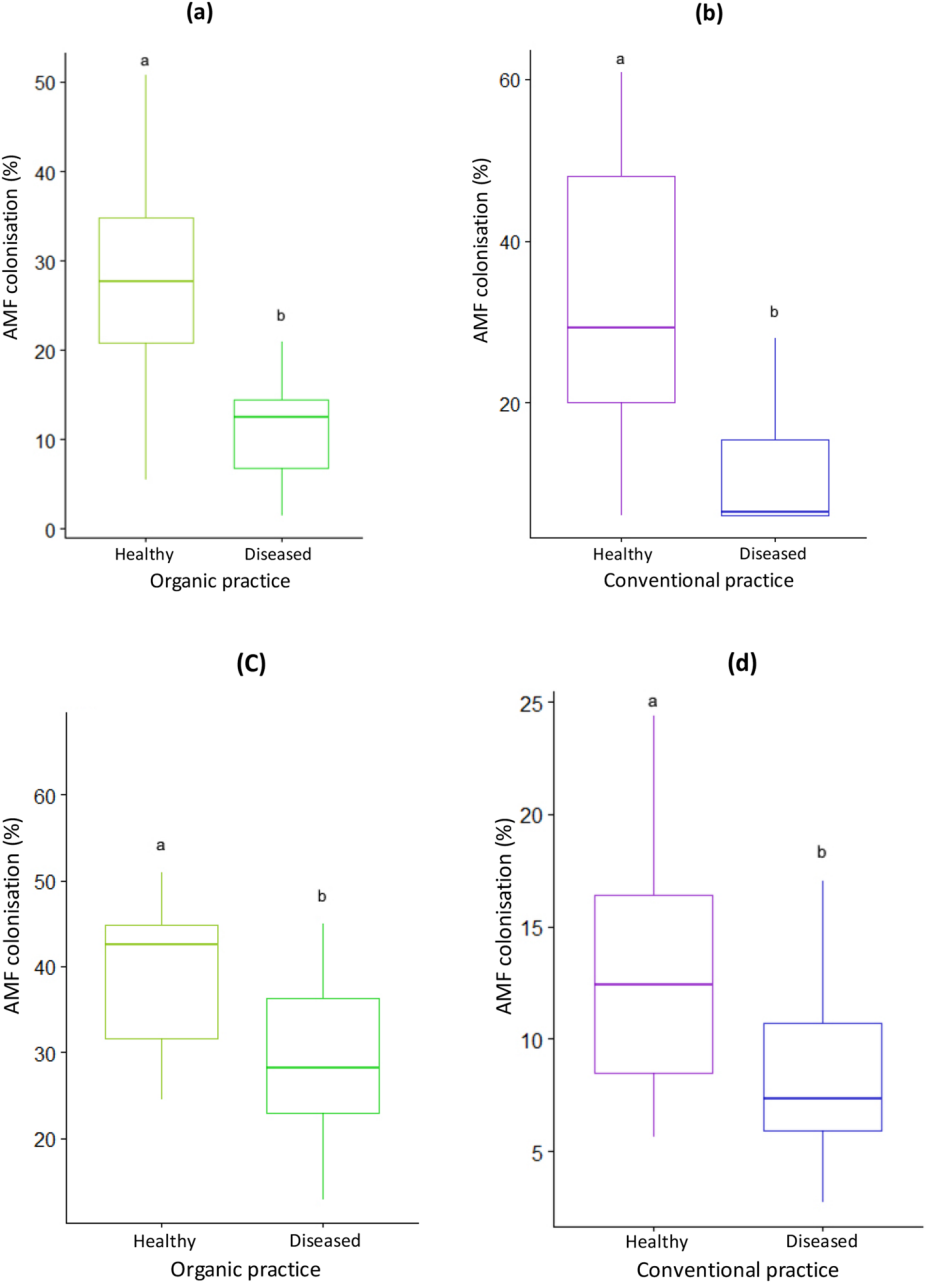
Arbuscular mycorrhizal fungal colonization in healthy and diseased plants of *L. angustifolia* (**a**, **b**) and *L. intermedia* (**c**, **d**) under different agricultural practices. Different letters indicate significant differences (Test Wilcoxon; *P* value < 0.05) according to the health status.

In lavender, whatever the agricultural practice, the mycorrhizal root colonization was significantly higher in the healthy plant compared to the diseased plant. It was 27% and 15% in the healthy and diseased plants, respectively, in organic practice and it was 30% and 5% in the healthy and diseased plants, respectively, in conventional practice (Fig. 1a,b). Variance partitioning indicated that the plant health status explained about 30% of the variability of the mycorrhizal root colonization in organic or conventional practices (Table S1). Similar patterns were observed in lavandin roots with a higher root colonization in the healthy plant compared to the diseased plant. In organic practice, it was 42% and 30% in the healthy and diseased plants, respectively and in conventional practice, it was 12% and 7% in the healthy plant and the diseased plant respectively. (Fig. 1c,d). However, the contribution of the plant health status in the variability in root colonization was lower compared to that observed in lavender roots and accounted approximately for 15% in each of the agricultural practices (Table S1).

### AMF diversity

**Figure 2:**
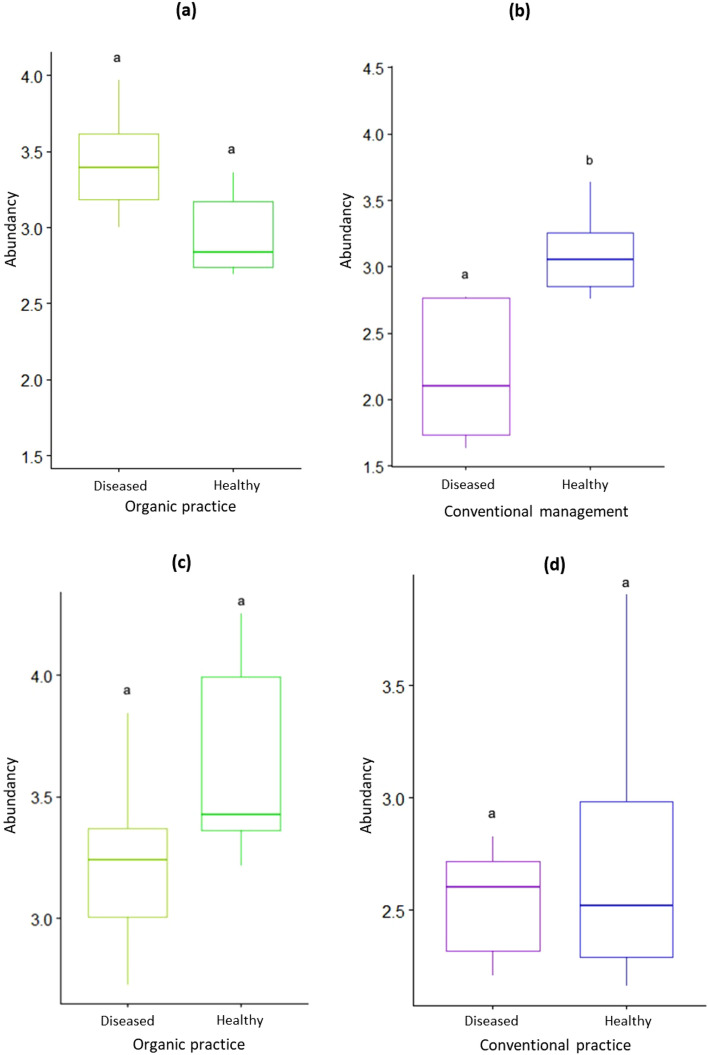
Abundance of AMF community in roots of healthy and diseased plants of *L. angustifolia* (**a**, **b**) and *L. intermedia* (**c**, **d**) under different agricultural practices. Different letters indicate significant differences according to the plant health state (Test Wilcoxon; *P* value < 0.05).

First, rarefaction analysis performed on the data indicated that the taxon accumulation curves reached saturation (Fig. S1). The AMF diversity was studied by focusing on the abundance and richness of fungal communities. Regarding the abundance of AMFs (corresponding to the abundance of number of sequences of each OTU), the results were different depending on the agricultural practice in lavender. We found an effect of the plant health status only in conventional practice which was reflected by a higher abundance in the healthy plants compared to the diseased plant (Fig. 2a,b). In lavandin, however, whatever the agricultural practice, the plant health status had no effect on the abundance of AMF (Fig. 2c,d).

**Figure 3:**
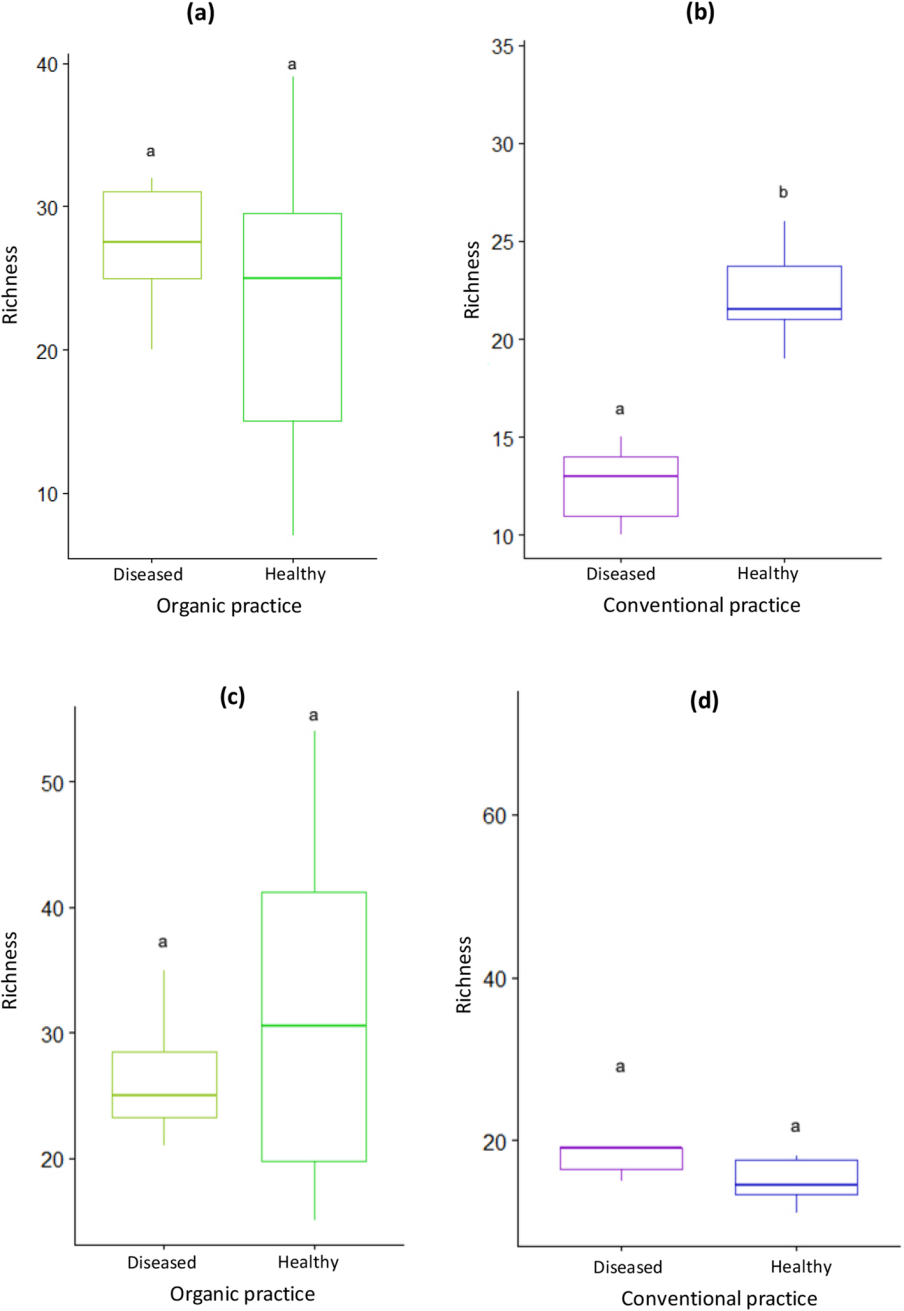
Richness of AMF community in roots of healthy and diseased plants of *L. angustifolia* (**a**, **b**) and *L. intermedia* (**c**, **d**) under different agricultural practices. Different letters indicate significant differences according to the plant health state (Test Wilcoxon; *P* value < 0.05).

Regarding the AMF richness, we observed a plant health status effect only in lavender in conventional practice. A greater AMF richness was observed in healthy plants compared to diseased plants (Fig. 3a–d).

### AMF distribution

**Figure 4:**
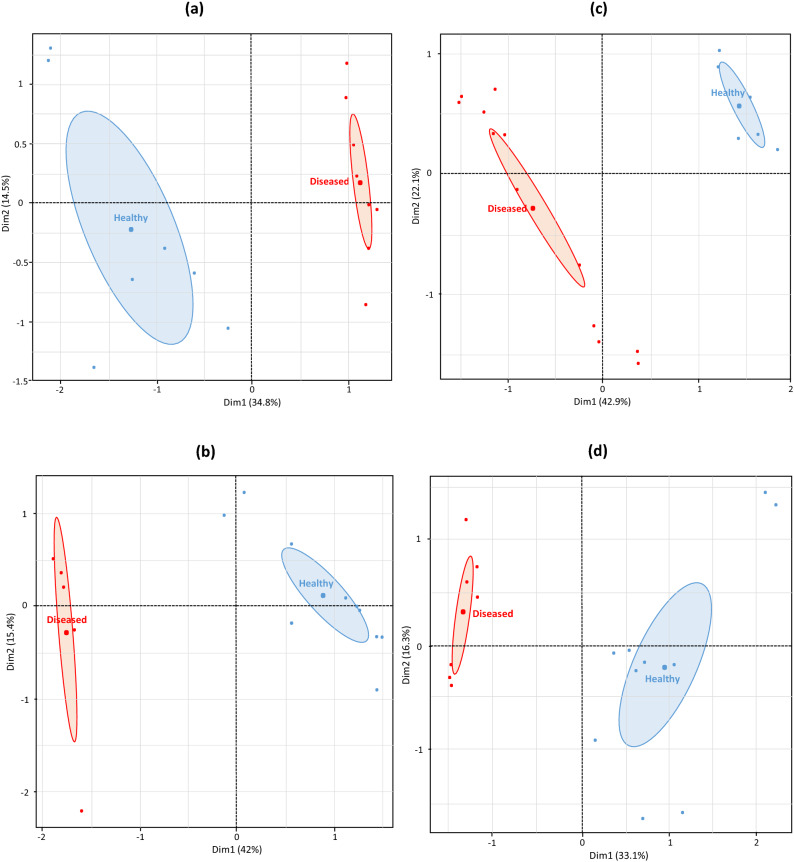
Multiple factor analysis of root AMF communities associated to *L. angustifolia* and *L. intermedia* roots. (**a**, **b**) Corresponded to *L. angustifolia* in organic and conventional practices, respectively; (**c**, **d**) corresponded to *L. intermedia* in organic and conventional practices, respectively.

In lavender roots, multiple factor analysis of the AM fungal communities, based on the abundance of AMF OTUs, identified two distinct AMF groups determined by the plant health status (Fig. 4a,b). In both agricultural practices, the first axis, which clearly separated the fungal communities according to the plant health status, accounted for 34.8% and 42% of the variation in organic and conventional practices, respectively. The second axis, which did not represent more than 16% of the variation, less clearly separated the AMF communities according to the plant health status. Permanova analysis indicated that the plant health status explained significant portion of the variance in fungal distribution (Table S2), 16% and 19% in organic and conventional practices, respectively. The other factors (abiotic or biotic) which influence AMF communities were represented by “residuals” and accounted for 84% and 81% in organic and conventional practices, respectively. By contrast to lavender, the plant health status had no significant effect on the structure of AMF communities in the lavandin roots whatever the agricultural practice (Fig. 4c,d and Table S2).

### AMF community composition

The global composition of AMF communities is displayed in Fig. S2. Globally, Glomeraceae dominated lavender and lavandin roots followed by unclassified Glomeromycetes. Claroideoglomeraceae, Acaulosporaceae and Gigasporaceae were present in small proportion and their distribution was variable depending on the plant species, the plant health status or the type of agricultural practice (Fig. S2).

**Figure 5:**
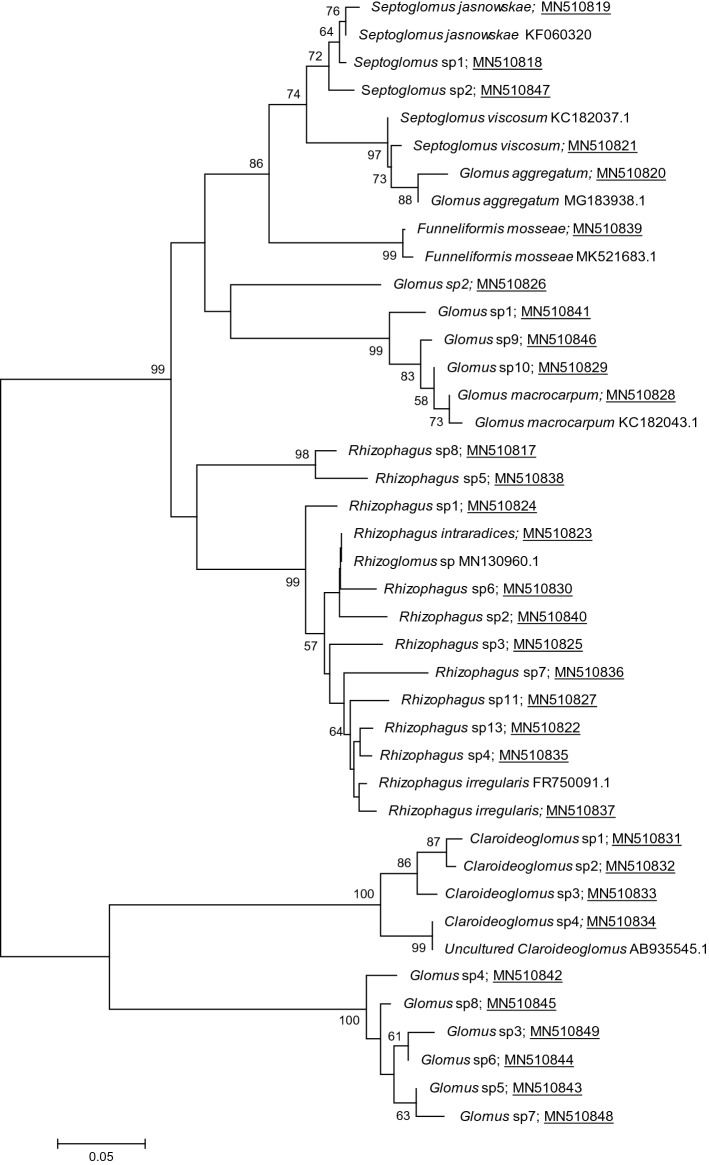
Phylogenetic analyses performed with the neighbour-joining method based on aligned sequences of 28S ribosomal RNA gene. Analyses were performed as described^40^. The AMF species from which the sequences (Genbank accession number in brackets) have been recovered from GenBank and used as reference sequences are indicated in red. The Genbank accession numbers from this work are underlined. Bootstrap values exceeding 50% are shown on the branches.

**Figure 6:**
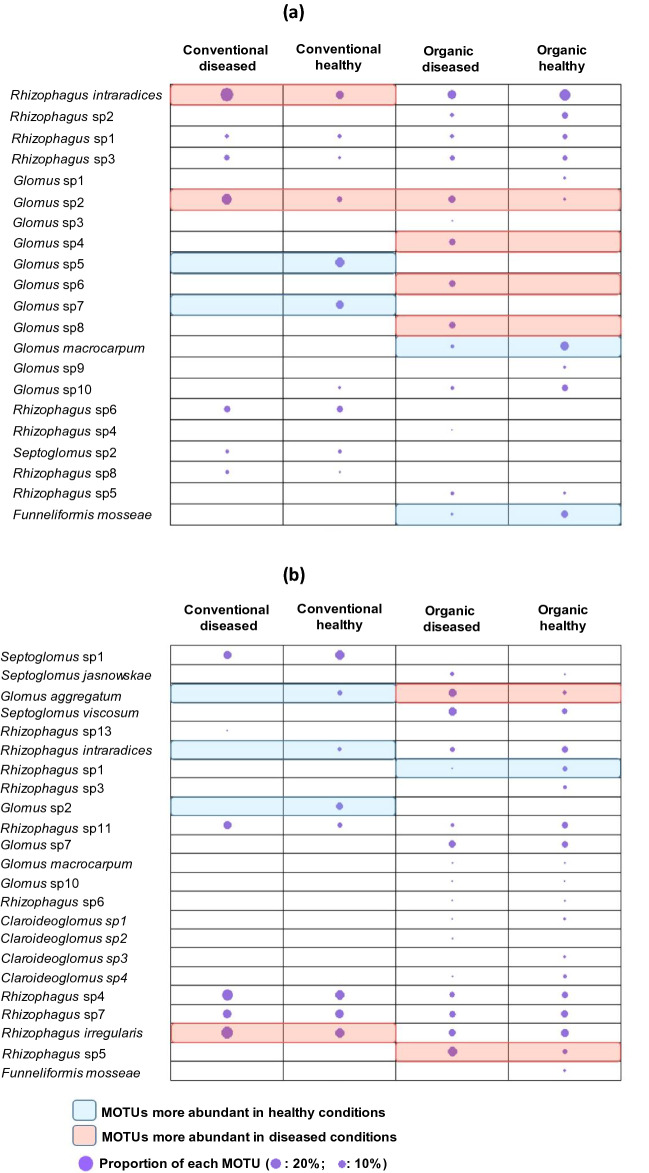
Composition of the most abundant AMF MOTUS in healthy and diseased plants of *L. angustifolia* (**a**) and *L. intermedia* (**b**) under different agricultural practices. The proportion of each MOTU (represented by circle) was compared between diseased and healthy conditions using a Chi-squared test (for conventional and organic agriculture, respectively).

The distribution of the most abundant AMF OTU i.e. present in at least 2/3 of the samples was also studied. First, their sequences, deposited in Genbank (MN510817-MN510849), were analyzed by phylogeny (Fig. 5) using the reference sequences available in the Genbank databases to confirm the taxonomic assignments and validate the specific delimitation of each MOTU. The most abundant AMF MOTUs found for each plant species were related to the plant health status in each agricultural practice. For example, in the lavender roots (Fig. 6a), 21 MOTUs were found with a variable distribution according to the plant health status in either agricultural practice. Among them, the most abundant MOTUs were *Rhizophagus intraradices* and *Glomus* sp2 in diseased lavender and *Glomus* sp5 and *Glomus* sp7 in healthy lavender under conventional practice on the one hand and *Glomus* sp2, *Glomus* sp4, *Glomus* sp6 and *Glomus* sp8 in diseased lavender and *Glomus macrocarpum* and *Funneliformis mosseae* in healthy lavender under organic practice on the other hand.

Likewise, in lavandin roots (Fig. 6b), 23 abundant MOTUs were found with variable patterns according to the plant health status in either agricultural practice. Among them, one MOTU (*Rhizophagus irregularis*) and 3 MOTUs (*Glomus aggregatum*, *Glomus* sp2 and *Rhizophagus intraradices*) were statistically more abundant in diseased and healthy lavandins respectively under conventional practice whereas 2 MOTUs (*Glomus aggregatum* and *Rhizophagus* sp5) and one MOTU (*Rhizophagus* sp1) were statistically more abundant in diseased and healthy lavandins respectively in organic practice.

## Discussion

Till now, studies that have investigated the plant health status effects on root AMF communities have been carried out on sites characterized by one agricultural management type^7,8^. These studies do not take into account the importance of different agricultural practices which are increasingly used to enhance crop yield and which are moreover described to influence structure and diversity of root AMF communities^9–13^. To bridge this gap, we investigated the specific effects of the plant health status of two Mediterranean crop plants, lavender and lavandin on the AMF colonization intensity and the structure of root AMF communities from organic and conventional fields affected by Phytoplasma infection. We assessed whether these effects differed according to the agricultural practices and were specific to each plant species.

We found an influence of the plant health status of both plant species on the intensity of root mycorrhizal colonization resulting in the establishment of a more effective symbiosis in the healthy plant compared to the diseased plant as demonstrated elsewhere^20,21^. Several factors could explain the implementation of symbiosis between AMF and host plants such as the root exudates^22^ or the ability of the host plant to supply C to AMF^1^. We suggested that lavender and lavandin produce root exudates whose quality and quantity, specific to each species, could vary according to the plant health status thus impacting AM symbiosis. It is also possible that diseased lavender and lavandin could reduce the C supply necessary for AMF development due to the modification in carbohydrate synthesis and transport as shown in phytoplasma-infected plants^23,24^. Plant health status effect was evidenced in both types of agricultural practices, however the fact that it was significantly greater in lavender than in lavandin (30% *vs* 15%, Table S1) could suggest the influence of the host plant identity on root AMF colonization^25–27^.

The mycorrhizal root colonization may be the reflection of the diversity of root AMF in host plants. Interestingly, we found that the plant health status impacted significantly the root AMF diversity by increasing the AMF abundance and richness in healthy lavender. However, this result, consistent with prior studies^7,8^, seemed to depend on the type of agricultural practices as it was seen only under the conventional practice whereas it was lacking in organic practice. We suggested that the plant health status and agricultural practices produce interactive effects on the diversity of root AMF communities in lavender. In lavandin however, consistent with the weaker effect of the plant health status on the root colonization, no effect of the plant health status was observed whatever the mode of agricultural practice. These contrasting results between lavender and lavandin may be related to the specificity of each species resulting probably from differences in root exudation patterns.

The survey of the distribution of root AMF communities partially confirms the patterns observed on fungal diversity, in particular the absence of plant health status effect on AMF communities associated with lavandin but its strong impact on the AMF associated with lavender as reported^8^. There are certainly other drivers, not measured in this study, which affect AMF communities including probably the agricultural practice, but in lavender, we did not detect the difference of the plant health status effect according to the agricultural practice as observed in the case of the diversity study. However, this result is not unusual as the sensitivity of the distribution or the diversity of AMF toward different drivers is not necessarily the same as reported elsewhere^28^.

The plant health status effects were also analyzed for the most abundant MOTUs present in at least 2/3 of the samples. Only MOTUs belonging to Glomeraceae and Claroideoglomeraceae families met these criteria. The plant health status effects were reflected more by differences in the MOTU abundance rather than the selection of specific MOTUs since some MOTUs considered as absent (Fig. 6a,b) are nevertheless present but in less than 1/3 of samples. Second, among the affected MOTUs, four different MOTUs for each species were significantly abundant in the roots of healthy lavender and lavandin in either agricultural practice. More interestingly, among selected MOTUs dominating in healthy plants, some such as *Funneliformis mosseae* or *Glomus macrocarpum* are described as being able to alleviate disease-related symptoms or plant stresses caused by abiotic conditions^4,29^. Plant health status may contribute to the selection of specific MOTUs in the roots of healthy plants, the amplification and use of which as an inoculum could probably help to strengthen or improve the resistance of plants against plant pathogens.

## Conclusions

Taken together, our results show the importance of plant health status on root AMF communities. This factor seems to promote in varying degrees the development of AMF symbiosis in plant roots and influence the diversity and composition of AM fungal communities. It may be affected by agricultural practices and depends on the host plant species identity. It is not excluded to think that the abundant MOTUs in the roots of healthy plants characterized here could contribute to the protection of these plants against Phytoplasma by immunizing the plant to a pathogen as well as other abiotic stresses.

## Materials and methods

### Site description

The study was conducted in four fields corresponding to two different agricultural practices of two species, the lavender (*L. angustifolia*, cultivar Rapido, seed derived populations) and the sterile hybrid lavandin (*L. intermedia*, cultivar Grosso, vegetatively propagated by cuttings) in the South-East of France. The coverage phytoplasma infections observed in these studied fields reached about 20—30% of the total plants. The description of management practices in organic and conventional fields for each plant species and soil characteristics are shown in Table 1. In both organic and conventional fields affected by Phytoplasma infection, two plant health status coexisted:—the asymptomatic plants defined as “healthy plants” showing no visible disease symptoms and—the symptomatic plants corresponded to “diseased plants” exhibiting typical symptoms of phytoplasma disease with yellowing and reduction of inflorescences on the whole plant^15^. These symptoms were examined by professional experts from CRIEPPAM (Manosque, France).

**Table 1:** Description of management practices in organic and conventional fields for each plant species, *L. angustifolia* and *L. intermedia* and soil characteristics.

Plant species	Management	GPS	Fertilizer applied	Pesticide use	Inter-row management	Organic matter (%)	Total carbone (%)	pH
L intermedia	Organic for 20 years	43° 54′ 5.15″ N 6° 11′ 3.34″ E	None	None	No tillage Plant cover (Fabaceae and Poaceae)	3.20 ± 0.10^a^	1.84 ± 0.01^a^	8.25 ± 0.05
	Conventional > 20 years	43° 52′ 23.2″ N	30:30:40 kg NPK ha^−1^	Herbicides and insecticides	Reduced tillage	2.70 ± 0.00^a^	1.57 ± 0.01^a^	8.25 ± 0.05
*L. angustifolia*	Organic for 4 years	44° 7′ 46.45″ N 5° 29′ 25.98″ E	None	None	Reduced tillage	5 ± 0.20^a^	2.90 ± 0.09^a^	8.2 ± 0.00^a^
	Conventional > 20 years	44° 8′ 8.12″ N 5° 28′ 1.38″ E	30:30:40 kg NPK ha^−1^	Herbicides and insecticides	Reduced tillage	4.2 ± 0.30^a^	2.46 ± 0.18^a^	8.25 ± 0.05

The herbicides and insecticides correspond to propyzamid, cycloxydim, fluroxypyr or amidosulfuron and chlorpyriphos-methyl for pest protection. The reduced tillage was performed on 5–10 cm depth. For each plant species, means with the same letter within columns are not significantly different (t-test; *P* < 0.05). Organic matter, total Carbone and pH were measured from sampled soils for each site by Aurea, France according to standard methods.

### Experimental approach

In area of 20 m × 20 m in each management field, in July 2017, we randomly collected 12 roots samples from the underground part of asymptomatic or symptomatic plants aged 2 years. All the resulting 96 roots samples (2 plant health status: healthy and diseased × 2 management types: organic and conventional × 2 plant species: lavender and lavandin × 12 replicates) were thoroughly rinsed with tap water and were used for both the determination of level of mycorrhizal colonization and DNA extraction.

### Level of mycorrhizal root colonization

Roots were stained as described^30^. The AMF colonization intensity was estimated under light microscopy^31^ and based on the MYCOCALC program (https://www.dijon.inra.fr/mychintec/Mycocalc-prg/download.html).

### DNA extraction, PCR amplification and illumina-based sequencing

For each plant species, the 12 root samples were pooled together two by two giving six samples for DNA extraction. The roots have been dried and ground with liquid nitrogen and 100 mg were used to extract DNA with the Fast prep DNA Spin kit (MPBIO, France) according to the manufacturer's instructions. The AMF-specific universal primers FLR3 and FLR4 was used to amplify the partial large subunit region^32,33^. The primers were extended by sample specific tags of 8 nt length to allow parallel sequencing of multiple samples. The PCR reactions were carried out as reported^34^ by adjusting the annealing temperature (58 °C) and the elongation time (30 s) according to the FLR3 and FLR4 primers. The amplicons were pooled for the sequencing by using equivalent molarities amongst amplicons. The library construction and sequencing (Illumina MiSeq 150-bp pair-end) were carried out by Fasteris (Geneve, Switzerland).

### Bioinformatic analysis

Reads assembly and primary filtering were done using the OBItools package^35^. Short (< 100 nt) or rare (occurency < 2) reads were removed, resulting in 138,593 unique sequences for 3,917,531 reads in total. Highly similar unique sequences were clustered into Molecular Operational Taxonomic Units (MOTUs) by computing pairwise similarities with Sumatra (OBItools package, https://metabarcoding.org/sumatra) and forming 98% similarity clusters^36^ with the Markov Cluster algorithm (MCL) classification process^37^. The ecotag function of OBItools was used to taxonomically assign the 3260 obtained MOTUs to an AMF database. It was generated from the r136 EMBL fungi database with an in silico PCR (function “ecoPCR”, OBItools package) using the FLR3 and FLR4 primers.

The R software 3.5.3 was then used to aggregate unique sequences belonging to the same MOTUs. From these sequences, we removed sequences of rare MOTUs (found in only one sample), sequences of the samples that have a Bray–Curtis scatter distance which exceeds 95% of their confidence interval and sequences of MOTUs not assigned to AMF clades. The dataset was then normalized by sample using Hellinger standardization method («descostand» function, package vegan). The final contingency table displays the relative abundancy (normalized read counts) of 190 MOTUs and 180 among the lavender and lavandin samples, respectively.

### Statistical analysis

Normality of the data was tested by Shapiro–Wilk test. Accordingly, the root colonization intensity (M %) and AMF diversity were analyzed by *Levene's test (for the homogeneity of variance) and* Student's t-tests or Wilcoxon test. The distribution of AMF communities was evaluated by Multiple Factor Analysis (MFA). The significance of the effect of plant health status on the root colonization intensity, diversity and structure of AMF communities were assessed by Permanova followed by Permutest. For each plant species, the dominant MOTUs (found in at least 2/3 of samples) were selected. Their composition was compared between diseased and healthy conditions—for conventional and organic agriculture respectively—using the Chi-squared test^38^, function «chisq.test») and Adjusted standardized residuals. All statistical analyses were performed using R package vegan^39^.

## Supplementary information

Supplementary Information.
