# Supplementary material for: Plant health status effects on arbuscular mycorrhizal fungi associated with Lavandula angustifolia and Lavandula intermedia infected by Phytoplasma in France
Source: Sci Rep. 2020 Nov 20;10:20305. doi: 10.1038/s41598-020-77240-6 (PMC7679420; doi:10.1038/s41598-020-77240-6)
Supplement: Supplementary file 1 — Supplementary Information. [file 41598_2020_77240_MOESM1_ESM.docx]

# Plant health status effects on arbuscular mycorrhizal fungi associated with *Lavandula angustifolia* and *Lavandula intermedia* infected by Phytoplasma in France

**Marie-Noëlle Binet ^1^ . Camille Marchal ^1^ . Justine Lipuma ^2^. Roberto Geremia ^1^ . Olivier Bagarri ^3^ . Bert Candaele ^4^ . Delphine Fraty ^4^ . Benjamin David ^2^ . Sophie Perigon ^1^ . Viviane Barbreau ^5^ . Bello Mouhamadou ^1 *^**

**SUPPLEMENTARY MATERIALS**

**Table S1.** Effects of the plant health status on the colonization intensity in the roots of healthy and diseased plants of *L. angustifolia* and *L. intermedia* under different agricultural practices. Values are the results of PERMANOVA and significance (****p* < 0.001, ***p* < 0.01, **p* < 0.05, ns; not significant).

**Table S2.** Effects of the plant health status on the distribution of AMF in the roots of healthy and diseased plants of *L. angustifolia* and *L. intermedia* under different agricultural practices. Values are the results of PERMANOVA and significance (****P* < 0.001, ***P* < 0.01, **P* < 0.05, ns, not significant).

***Lavandula augustifolia***

***Lavandula intermedia***

**
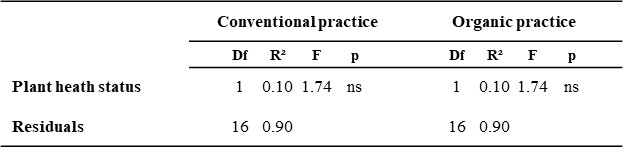
**

**Fig. S1** Rarefaction curves from the number of sequences from *L. angustifolia* and *L. intermedia* samples


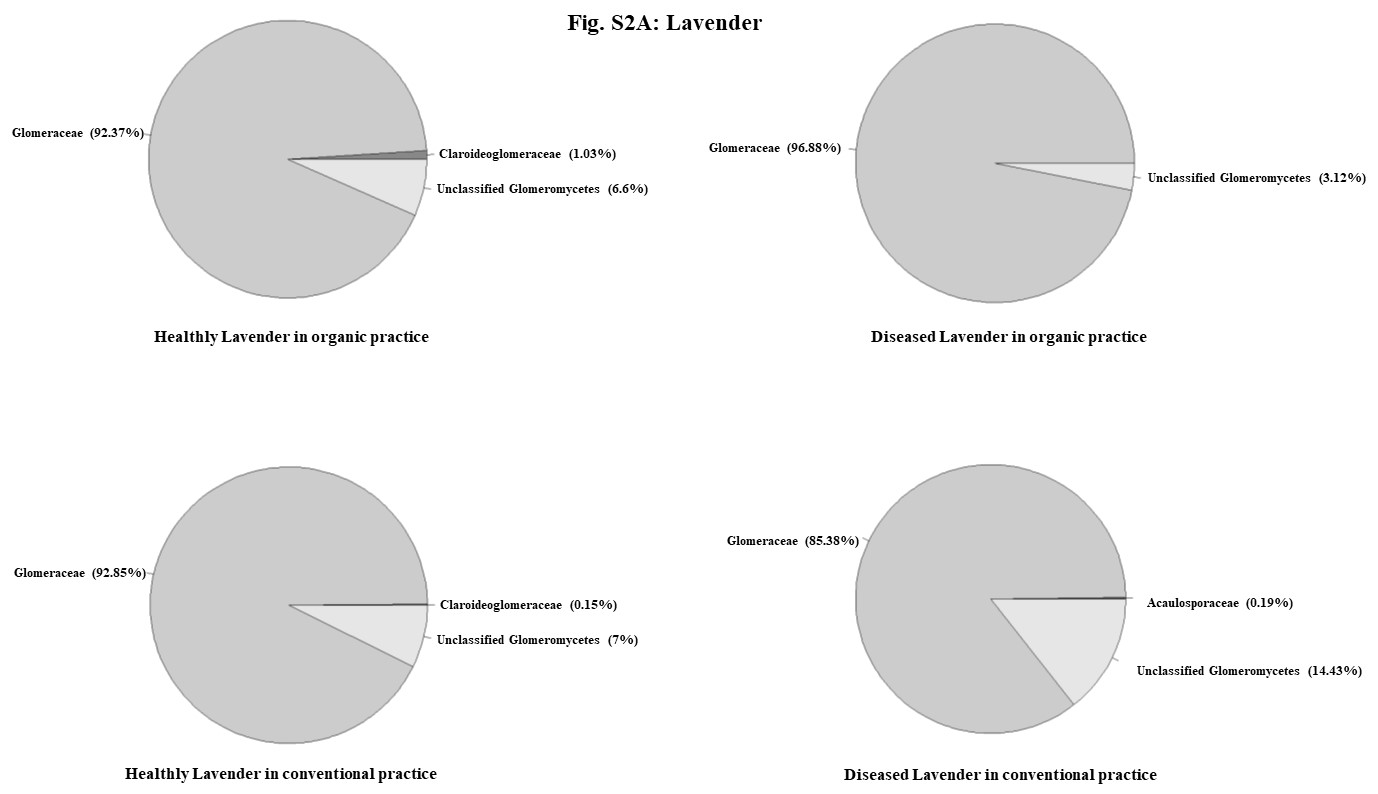


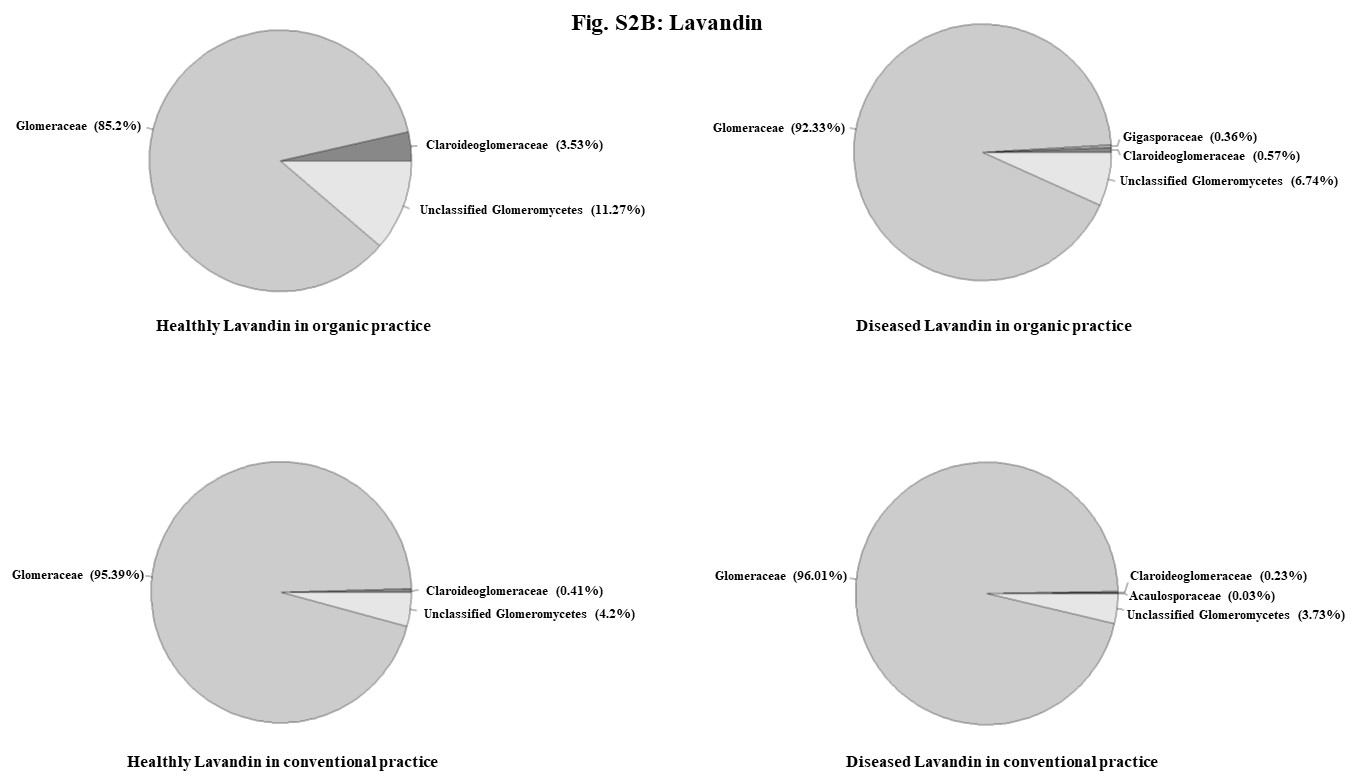


**Fig. S2** Distribution of AMF communities associated to lavender and lavandin roots.
